# A Meta-analysis of Cognitive Functioning in Intimate Partner Violence Perpetrators

**DOI:** 10.1007/s11065-023-09628-w

**Published:** 2023-12-12

**Authors:** Ángel Romero-Martínez, Carolina Sarrate-Costa, Luis Moya-Albiol

**Affiliations:** Department of Psychobiology, University of Valencia, Avenida Blasco Ibañez 21, Valencia, 46010 Spain

**Keywords:** Intimate partner violence, Men, Meta-analysis, Neuropsychology, Violence

## Abstract

Numerous psychologists have shown interest in applying neuropsychological tests to study intimate partner violence (IPV) perpetrators and gain a further understanding of the underlying nature of this type of violence. However, up until now, no meta-analysis has drawn on all the available scientific literature to calculate whether consistent differences exist between the neuropsychological performance of IPV perpetrators and other samples of men (non-violent men, IPV perpetrators with drug misuse, and other men with criminal history). The aim of this study was to carry out this calculation and also measure whether neuropsychological performance explained IPV perpetration. We conducted a meta-analysis following the Preferred Reporting Items for Systematic Reviews and Meta-Analyses (PRISMA) criteria. After initially identifying 7243 sources, we eventually included a total of 25 publications. The number of studies included for effect size calculation in each cognitive domain ranged from two to nine. Taking solely into account studies comparing IPV perpetrators with non-violent men, our findings indicate that both IPV perpetrators who misuse drugs and those who do not exhibit worse neuropsychological functioning compared to non-violent men. These differences range from moderate to large for working memory, switching attention, cognitive flexibility, planning abilities, and phonemic fluency. However, while low functioning in response IQ was only observed in IPV perpetrators without drug misuse, continuous attention performance only differed in IPV perpetrators with drug misuse. It should be noted that most conclusions were consistent. In addition, the comparison between IPV perpetrator subsamples and other types of criminal convictions only revealed differences in switching attention, with IPV perpetrators presenting worse abilities than the rest of the subsamples. Finally, we also found some support for significant associations between neuropsychological performance and both physical and psychological IPV perpetration. This meta-analysis is a significant contribution that will help inform future clinical strategies for the early detection of cognitive needs. It will also guide the implementation of new or complementary intervention programs.

**Supplementary Information:**

The online version contains supplementary material available at 10.1007/s11065-023-09628-w.

## Introduction

Numerous psychologists have shown interest in applying neuropsychological tests to study violence and gain a deeper understanding of its underlying nature or etiology (Golden et al., 1996; Richman et al., 1999). Using these instruments helps overcome the limitations of self-reports, which are commonly used in psychology but are limited by factors such as social desirability, lack of honesty in responses, and difficulties with introspective ability, among others (Richman et al., 1999). Therefore, they allow complementing the conclusions from self-reports to clarify how these men process information (Howlett et al., 2021; Snyder et al., 2021).

Neuropsychological hypotheses that try to explain violence are framed within social information processing theories (Dodge & Crick, 1990; Dodge & Coie, 1987). That is, they conceive the onset of violence as a result of failures in cognitive processing and misinterpretation of surrounding information and inner states that allow properly coping with these signals. This diminished ability to process information has been linked to memory failures, attention deficits, and low cognitive flexibility in adapting to a demanding context, among others (Hunter et al., 2022). These failures to process information increase the likelihood of only attending to salient stimulus (e.g., hostile, potentially damaging, those with emotional content, novel stimuli), while neglecting the rest of the surrounding information (Tegelbeckers et al., 2015) and making inadequate decisions based on failures when processing basic sources of information. This misunderstanding makes people more likely to respond with violence in specific situations (Golden et al., 1996; Ogilvie et al., 2011; Reinharth et al., 2014; Sedgwick et al., 2017), particularly, when these deficits are combined with other hostile cognitive schemas and attributes (i.e., pathological jealousy, inappropriate feelings toward themselves and toward their partners). Therefore, cognitive deficits in combination with hostile schemas or when under the effect of drugs might facilitate inadequate responses such as antisocial and violent behaviors, especially under emotionally uncomfortable and uncertain circumstances (Martel, 2019; Siever, 2008).

The employment of neuropsychological tests might provide critical insight regarding the main causes of violence perpetration. In this regard, early detection and monitoring of these cognitive deficits in certain violent individuals can have major implications for treatment implementation (Casaletto & Heaton, 2017; Sherman et al., 2017). In the past 20 years, the use of these techniques in the study of domestic violence, specifically in evaluating the cognitive/neuropsychological characteristics of men convicted of intimate partner violence (IPV) against women, has expanded our knowledge of the main causes that contribute to this type of violence (Pinto et al., 2010). However, it cannot be stated that these are the main underlying factors of IPV perpetration given that cognitive dysfunctions and misinterpretation tend to be closely related to drug misuse (Capaldi et al., 2012; Romero-Martínez & Moya-Albiol, 2013). Thus, to properly understand the existence of cognitive deficits in IPV perpetrators, the role of drug misuse would need to be controlled.

Despite the interest and importance of traditional narrative and systematic reviews to understand complex phenomenon such as violence, they have not necessarily focused on quantitative inconsistencies in terms of findings, methodological quality assessment, or the limitations inherent to the type of designs employed in empirical research (Greenhalgh et al., 2018; Pae, 2015). Not having this information might offer a partial or distorted vision of the neuropsychological functioning of IPV perpetrators’ cognitive functioning, underestimating or even overestimating certain deficits with clinical relevance. In other words, the absence of a quantitative perspective (accounting for group differences) might neglect the clinical relevance of certain cognitive domains that could be used as guidance for clinical experts. This emphasizes the need for conducting a meta-analysis to complement previous findings and conclusions regarding the cognitive functioning of IPV perpetrators. In other words, there is a need for summarizing relevant literature by estimating the effect sizes of these differences (Uman, 2011).

To the authors’ knowledge, it is necessary to conduct a meta-analysis of the literature in order to determine if differences in neuropsychological performance can be used to distinguish men convicted of IPV (or IPV perpetrators) from other groups of men, especially, from non-violent men, IPV perpetrators with drug misuse (a potential confounding factor underlying IPV perpetration (Capaldi et al., 2012)) and other types of criminal convictions. This is relevant given that many authors claim that neuropsychological/cognitive deficits were the main underlying factors of violence perpetration. In addition, it would help clinicians design intervention programs tailored to these men (Sedgwick et al., 2017). We, therefore, hypothesized that IPV perpetrators, especially those with drug misuse, would show statistically discernable differences in several cognitive domains, particularly in executive functioning, compared to non-violent men. Second, we tested whether neuropsychological deficits in IPV perpetrators would explain IPV proneness (i.e., physical, and psychological). We further hypothesized that a broader extension of these neuropsychological deficits would imply higher levels of all types of IPV.

## Methods

### Search Strategy

This meta-analysis was conducted in accordance with the guidelines established in the Preferred Reporting Items for Systematic Reviews and Meta-Analyses (PRISMA) (Hutton et al., 2015) and was registered in OSF (Registration DOI: https://doi.org/10.17605/OSF.IO/PBHFD). In the first place, records were accessed from six digital databases: PubMed, PsycINFO, Psicodoc, Web of Science, Dialnet, and Cochrane Library. This was in accordance with the recommendations stated by Bramer et al. (2017) to conduct a meta-analysis or systematic review by employing a minimum of three different databases. Moreover, when possible, database filters were applied to reduce the number of entries; for example, some of them gave us the opportunity to remove entries including the following: “books,” “graduate theses and dissertations,” “case reports,” “animal studies,” “literature review,” “meta-analysis,” and other languages different from “English and/or Spanish.” However, we decided not to impose date restrictions to cover as many entries as possible. Last, to account for as much research as possible, searches in these digital databases were complemented by hand searching for additional articles that met the eligibility criteria but were not included in the above-mentioned digital databases (articles in press, papers not indexed in these digital databases, etc.). Two entries were obtained through this procedure. We used ResearchGate and Google Scholar for this purpose.

The search for all published studies was performed from May to July 2022.

The algorithm employed to conduct this review was as follows: ((*cognitive* **OR** *neuropsychology* **OR** *neuropsychological* **OR** *neurocognitive* **OR** *executive functioning* **OR** *deficits* **OR** *executive dysfunction* **OR** *deficits*) **AND** (*intimate partner violence* **OR** *maritally violent men* **OR** *domestic violence offenders* **OR** *spousal violence* **OR** *domestic violence* **OR** batterers)).

### Inclusion Criteria

**Fig. 1:**
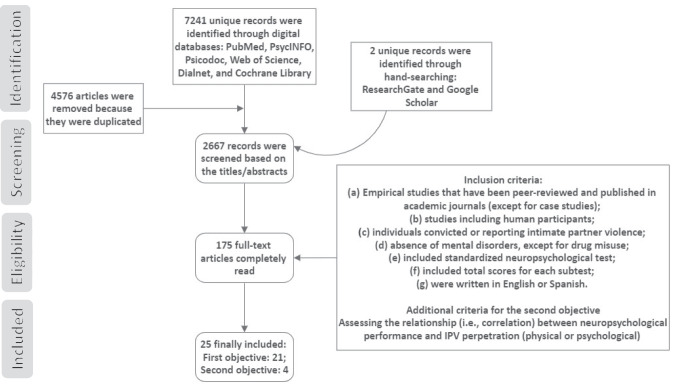
PRISMA flow chart of literature search with inclusion criteria

In accordance with McKenzie et al. (2022), the following selection criteria for conducting systematic reviews and meta-analysis for all the included studies were formulated: (a) empirical studies that have been peer-reviewed and published in academic journals (except for case studies); (b) studies including human participants; (c) individuals convicted of or reporting intimate partner violence (measured with validated scales such as Conflict Tactics Scales (CTS) (Straus, 1979) or Revised Conflict Tactics Scales (CTS2) (Straus et al., 1996); (d) absence of mental disorders (i.e., schizophrenia, depression, bipolar disorders, etc.), except for substance use disorders or drug misuse; (e) studies including standardized neuropsychological tests; (f) studies including total scores for each subtest (e.g., total score after adding direct and indirect subscales); and (g) studies written in English or Spanish. Moreover, we considered the following criterion only for the second objective of this study: assessing the relationship (i.e., correlation) between neuropsychological performance and IPV perpetration (physical or psychological) (PRISMA flow diagram; Fig. 1).

### Exclusion Criteria

First, empirical studies which employed neuropsychological tests but did not provide direct data (e.g., published, as supplementary material or provided after asking for data) to calculate the effect sizes were removed from this review. Second, studies which established subgroups of IPV perpetrators without clearly stating the criteria employed were also removed. Third, we removed articles initially included in which the authors calculated a total score based on different neuropsychological tests but did not include direct scores of each test separately. Last, studies that did not provide enough information, which would question the quality and replicability of this study, were also removed.

### Quality Assessment

Due to the broad heterogeneity in the studies’ methodological characteristics, we considered it particularly interesting to assess the quality of included comparative empirical studies by providing a supplementary table summarizing key points of their designs. We followed the recommendations stated in the National Institutes of Health (NIH) (2021) to assess the potential risk of bias when interpreting the data in non-randomized cross-sectional studies. Based on previous literature establishing quality criteria for non-randomized and randomized studies, we included twelve statements that would be answered with a dichotomous answer (Yes/No) indicating whether each criterion was met or not. Based on the answers, we indicated whether studies presented a low, moderate, or high-risk of bias. Studies which neglected two or less than two criteria were classified as “low risk of bias,” those which ranged from three to five were qualified as “moderate risk of bias,” and those neglecting six or higher than six criteria were classified as “high risk of bias” (See Supplementary Table 1).

Based on recommendations stated by the National Institutes of Health (NIH) (2021) to evaluate the quality of cross-sectional studies and our previous experience in this field, we considered it appropriate to define the following statements to assess risk of bias of the research included in this meta-analysis:

#### Objectives and Hypotheses

- Objectives clearly defined based on previous scientific literature by citing references and specifying the main outcomes (e.g., providing domain-specific processes).
- Hypotheses clearly defined based on previous scientific literature by citing references and specifying the main outcomes (e.g., providing domain-specific processes).

#### Sample

- Inclusion criteria specified and clearly stated.
- Authors followed a similar selection process for all samples included (when applicable).
- Offered enough information about experimental mortality.
- Calculation of sample size included.
- Sample size equal to or higher than 50 participants (total or per group when authors compare neuropsychological functioning or association with IPV).

#### Procedure

- Employed reliable instruments by providing psychometric properties of their study or previously published references.
- Clearly defined the procedure (e.g., time frame, experimental phases).

## Results

- Absence of differences between groups in potential confounding variables (e.g., number of participants per group, sociodemographic differences such as age, educational level, among others).
- Covariated potential confounding variables.
- Provided mean values and standard deviations to conduct the meta-analysis or, if missing, were obtained from authors upon request.

### Data Extraction and Analysis

Article selection was carried out by two researchers, with an interrater agreement above 90%. Any possible discrepancies between the two raters were discussed, and the third author of the manuscript was consulted to clarify whether critical points met the inclusion criteria.

### Data Analysis

To calculate the effect sizes and address the first aim of this study, we employed Review Manager 5.4 software from the Cochrane Collaboration (Higgins et al., 2022). Accordingly, this study employed random-effects modeling and included the calculation of the total effect (*Z* value) for standardized mean difference (SMD) across studies with 95% confidence intervals (95% CI). Moreover, heterogeneity across studies was calculated by including Tau^2^, Chi^2^, and *I*^2^.

Regarding the second aim, the Meta-Essentials for correlational data 1.5 (for further details see Suurmond et al., 2017) was used for the correlation between neuropsychological performance and IPV perpetration (assessed with the physical assault and psychological aggression subscales f the CTS or CTS2), providing the same statistical descriptors as those stated above. These IPV subscales were continuous measurements which assessed the frequency of these behaviors during past year. The response to each item ranged from the absence of these type of behaviors to more than 20 times during this period, with higher scores indicating a higher presence of these types of IPV (Straus, 1979; Straus et al., 1996).

## Results

### Selection of Studies

The database search yielded 7241 sources, and an additional 2 articles were identified through other sources (i.e., hand-searching). From this total of publications retrieved from the search of the digital databases, 4576 articles were removed because they were duplicated, and 2705 were excluded based on the title or abstract because they were not relevant to the main objective of this review. Hence, from 175 full-text articles completely read, only 25 were finally included (see the flowchart described in Fig. 1). A total of 21 articles were selected to address the first aim of this study, and data for the second objective came from 4 studies (Supplementary Table 2).

It should be noted that we only described results for cognitive domains that existed in a minimum of two different articles which measured them. This was in line with the recommendations pointed out by Ryan (2016) for conducting meta-analysis.

#### Working Memory

The assessment of working memory (visuospatial) performance revealed a significant difference such as that IPV perpetrators (without drug misuse) scored below non-violent men in the working memory test (5 studies: SMD = −0.48, 95% CI [−0.67, −0.29]; test for overall effect: *Z* = 4.86, *p* < 0.00001). Heterogeneity assessment revealed that conclusions across studies were relatively homogeneous given that there were no significant differences (Heterogeneity: Tau^2^ = 0.00, Chi^2^ = 2.48, *p* = 0.65; *I*^2^ = 0%) (Table 1a).

**Table 1:** Working memory performance in IPV perpetrators without (a) and with drug misuse (b) compared to non-violent men

(a)									
**Study**	**Test**	**IPV perpetrators**			**Controls**			**Weight**	**Std. mean difference**
		**Mean**	**SD**	**Sample size**	**Mean**	**SD**	**Sample size**		**IV, random, 95% CI**
Amaoui et al. (2022)	Letters and numbers	7.24	3.09	25	9.1	2.67	29	12.4%	−.64 [−1.19, −.09]
Easton et al. (2008)	Spatial working memory (computerized)	1.3	0.75	9	1.4	0.69	7	3.8%	−.13 [−1.12, .86]
Romero-Martínez et al. (2021b)	SSP (CANTAB)	5.18	1.66	51	6.23	1.6	39	20.4%	−.64 [−1.06, −.21]
Romero-Martínez et al. (2022a, b)	Digit span WAIS-III	14.05	4.19	120	15.46	3.82	82	46.7%	−.35 [−.63, −.06]
Vitoria-Estruch et al. (2018)	Spatial location WMS-III	15.52	4.0	35	17.81	3.35	37	16.7%	−.62 [−1.09, −.14]
Total (95% CI)				240			194	100.0%	−.48 [−.67, −.29]
Heterogeneity: Tau^2^ = .00; Chi^2^ = 2.48, *p* = .65; *I*^2^ = 0%; Test for overall effect: *Z* = 4.86 (*p* < .00001)									
**(b)**									
**Study**	**Test**	**IPV perpetrators + drug misuse**			**Controls**			**Weight**	**Std. mean difference**
		**Mean**	**SD**	**Sample size**	**Mean**	**SD**	**Sample size**		**IV, random, 95% CI**
Easton et al. (2008)	Spatial working memory	1.8	1.4	9	1.4	0.69	9	8.4%	0.35 [−0.59, 1.28]
Romero-Martínez et al. (2019c)	Spatial location WMS-III	15.47	3.67	63	17.62	3.38	39	28.8%	−0.60 [−1.01, −0.19]
Romero-Martínez et al. (2022a, b)	Digit span WAIS-III	13.78	3.89	104	15.46	3.82	82	42.3%	−0.43 [−0.73, −0.14]
Vitoria-Estruch et al. (2018)	Spatial location WMS-III	15.41	3.28	28	17.81	3.35	37	21.9%	−0.71 [−1.22, −0.21]
Total (95% CI)				204			167	100.0%	−0.48 [−0.75, −0.21]
Heterogeneity: Tau^2^ = .02; Chi^2^ = 4.25, *p* = .24; *I*^2^ = 29%; Test for overall effect: *Z* = 3.49 (*p* = .0005)									

When we compared the performance of IPV perpetrators with drug misuse and non-violent men in terms of working memory, conclusions were the same. That is, IPV perpetrators with drug misuse presented lower working memory performance than non-violent men (4 studies: SMD = -0.48, 95% CI [−0.75, −0.21]; test for overall effect: *Z* = 3.49 (*p* = 0.0005)). Moreover, the low heterogeneity across included studies could be verified given that the *I*^2^ was lower than 40% (Heterogeneity: Tau^2^ = 0.02, Chi^2^ = 4.25, *p* = 0.24); *I*^2^ = 29%) (Table 1b).

Last, the analysis of SD averages across studies in both cases could be defined as “medium,” given that they were close to the value of 0.05. In this sense, group differences were similar in both cases.

#### Attention

Calculating the ability to switch attention across studies revealed that IPV perpetrators without drug misuse tend to present serious difficulties to change their attentional focus compared to non-violent men (9 studies: SMD = 0.45, 95% CI [0.27, 0.62]; test for overall effect: *Z* = 5.09 (*p* = 0.00001), with this conclusion being consistent across studies (Heterogeneity: Tau^2^ = 0.00, Chi^2^ = 5.30, *p* = 0.73; *I*^2^ = 0%) (Table 2a).

**Table 2:** Switching attention performance in IPV perpetrators without (a) and with drug misuse (b) compared to non-violent men

**(a)**									
**Study**	**Test**	**IPV perpetrators**			**Controls**			**Weight**	**Std. mean difference**
		**Mean**	**SD**	**Sample size**	**Mean**	**SD**	**Sample size**		**IV, random, 95% CI**
Amaoui et al. (2022)	Spatial Stroop Swith RC across	−78.14	66.06	26	−111.12	54.84	28	9.9%	.54 [−0.01, 1.08]
Cohen et al. (2003)	TMT B time	82.7	36.9	41	64.5	24.4	20	10.0%	.54 [−.01, 1.08]
Easton et al. (2008)	TMT B time	69.6	32.1	9	50.1	5.7	7	2.8%	.75 [−.28, 1.78]
Romero-Martínez et al. (2019b)	AST switch	−76.09	112.72	89	−142.44	116.86	39	20.0%	.58 [.20, .96]
Romero-Martínez et al. (2021b)	AST switch	−87.8	123.67	51	−142.44	116.86	39	16.5%	.45 [.03, .87]
Stanford et al. (2007)	TMT B time	49.0	11.1	18	48.4	9.5	18	6.9%	.06 [−.60, .71]
Vitoria-Estruch et al. (2018)	AST switch	−134.42	123.81	35	−143.56	119.83	37	13.8%	.07 [−.39, .54]
Westby and Ferraro (1999)	TMT B time	61.36	22.37	38	50.82	16.35	38	14.0%	.53 [.07, .99]
Salas Picón and Cáceres Duran (2017)	TMT B time	1.47	0.943	17	0.88	0.857	17	6.2%	.64 [−.05, 1.33]
Total (95% CI)				324			243	100.0%	.45 [.27, .62]
Heterogeneity: Tau^2^ = .00; Chi^2^ = 5.30, df = 8 (*p* = .73); *I*^2^ = 0%; test for overall effect: *Z* = 5.09 (*p* < .00001)									
(b)									
**Study**	**Test**	**IPV perpetrators + drugs**			**Controls**			**Weight**	**Std. mean difference**
		**Mean**	**SD**	**Sample size**	**Mean**	**SD**	**Sample size**		**IV, random, 95% CI**
Easton et al. (2008)	TMT B time	94.9	25.8	9	50.1	5.7	7	12.5%	2.13 [.83, 3.44]
Romero-Martínez et al. (2021a)	TMT B time	133.62	83.57	47	77.88	61.38	41	46.5%	.75 [.31, 1.18]
Vitoria-Estruch et al. (2018)	AST switch	−55.9	80.57	28	−143.56	119.83	37	41.0%	.83 [.31, 1.34]
Total (95% CI)				84		85		100.0%	.95 [.44, 1.46]
Heterogeneity: Tau^2^ = .10; Chi^2^ = 3.94, *p* = .14; *I*^2^ = 49%; test for overall effect: *Z* = 3.67, *p* = .0002									

The comparison between IPV perpetrators with drug misuse and non-violent men revealed significant and marked differences between both groups in terms of switching attention (3 studies: SMD = 0.95, 95% CI [0.44, 1.46]; test for overall effect: *Z* = 3.67 (*p* = 0.0002), with a moderate heterogeneity across studies (Heterogeneity: Tau^2^ = 0.10, Chi^2^ = 3.94, *p* = 0.14; *I*^2^ = 49%) (Table 2b).

Whereas the calculation of the first comparison revealed a “moderate” difference, the second comparison revealed a “large” effect. Nonetheless, the heterogeneity across studies was higher in the second comparison.

The analysis of group differences in terms of continuous attention performance revealed that there were no significant differences between IPV perpetrators without drug misuse and non-violent men (5 studies: SMD = 0.12, 95% CI [−0.19, 0.43]; test for overall effect: *Z* = 0.86, *p* = 0.39). This conclusion was consistent across all included studies (Heterogeneity: Tau^2^ = 0.03, Chi^2^ = 6.30, *p* = 0.18;* I*^2^ = 37%) (Table 3a).

**Table 3:** Continuous attention performance in IPV perpetrators without (a) and with drug misuse (b) compared to non-violent men

**(a)**									
**Study**	**Test**	**IPV perpetrators**			**Controls**			**Weight**	**Std. mean difference**
		**Mean**	**SD**	**Sample size**	**Mean**	**SD**	**Sample size**		**IV, random, 95% CI**
Amaoui et al. (2022)	Go-No Go RT	−1.31	17.49	25	8.62	22.79	29	16.9%	−.48 [−1.02, .07]
Cohen et al. (1999)	ARCPT: Inconsistency Index	8.8	3.4	39	7.8	3.1	63	26.6%	.31 [−.09, .71]
Cohen et al. (2003)	Go No Go errors	.62	1.0	41	.3	0.5	20	14.8%	.36 [−.18, .90]
Easton et al. (2008)	CPT errors	36.1	27.5	9	33.8	15.9	7	4.4%	.09 [−.89, 1.08]
Romero-Martínez et al. (2022a, b)	CPT-III errors	28.84	22.2	120	25.81	15.62	82	54.2%	.15 [−.13, .43]
Total (95% CI)				234			201	100.0%	.12 [-.15, .38]
Heterogeneity: Tau^2^ = .03; Chi^2^ = 6.30, df = 4 (*p* = .18); *I*^2^ = 37%; test for overall effect: *Z* = .86 (*p* = .39)									
(b)									
**Study**	**Test**	**IPV perpetrators + drugs**			**Controls**			**Weight**	**Std. mean difference**
		**Mean**	**SD**	**Sample size**	**Mean**	**SD**	**Sample size**		**IV, random, 95% CI**
Easton et al. (2008)	CPT errors	385.9	75.0	9	330.6	75.8	7	8.1%	.69 [−.33, 1.72]
Romero-Martínez et al. (2019c)	AST correct responses	84.34	17.15	63	92.03	6.73	39	23.1%	−.54 [−.95, −.13]
Romero-Martínez et al. (2021a)	D2 total effectiveness	323.51	104.28	47	421.37	88.33	41	21.6%	−1.00 [−1.44, −.55]
Romero-Martínez et al. (2022a, b)	CPT-III Hits rate	278.13	18.71	120	285.68	5.31	82	28.0%	−.51 [−.79, −.22]
Vitoria-Estruch et al. (2018)	AST correct responses	86.48	10.96	28	93.47	6.56	37	19.2%	−.79 [−1.30, −.28]
Total (95% CI)				267			206	100.0%	−.64 [−.83, −.45]
Heterogeneity: Tau^2^ = .00; Chi^2^ = 4.13, df = 4 (*p* = .39); *I*^2^ = 3%; test for overall effect: *Z* = 6.49 (*p* = .00001)									

However, the assessment of group differences between IPV perpetrators with drug misuse and non-violent men revealed significant differences across studies (5 studies: SMD = −0.64, 95% CI [−0.84, −0.45]; test for overall effect: *Z* = 6.49 (*p* < 0.00001), with this conclusion being homogeneous (Heterogeneity: Tau^2^ = 0.00, Chi^2^ = 4.13, *p* = 0.39; *I*^2^ = 3%) (Table 3b). Furthermore, the average effect size across studies might allow us to conclude the existence of a moderate difference between IPV perpetrators with drug misuse and non-violent men.

#### Executive Functioning

##### Cognitive Flexibility

Analyses of neuropsychological performance, concretely those employing perseverative errors subscales of the WCST, revealed a significant difference such that IPV perpetrators without drug misuse committed more perseverative errors than non-violent men (9 studies: SMD = 0.66, 95% CI [0.48, 0.84]; test for overall effect: *Z* = 7.24 (*p* < 0.00001), with this conclusion being relatively consistent across studies (Heterogeneity: Tau^2^ = 0.02, Chi^2^ = 11.50, *p* = 0.17; *I*^2^ = 30%) (Table 4a).

**Table 4:** Cognitive flexibility performance in IPV perpetrators without (a) and with drug misuse (b) compared to non-violent men

**(a)**										
**Study**	**Test**	**IPV perpetrators**			**Controls**				**Weight**	**Std. mean difference**
		**Mean**	**SD**	**Sample size**	**Mean**		**SD**	**Sample size**		**IV, random, 95% CI**
Cohen et al. (1999)	WCST perseverative errors	21.4	14.8	39	13.9		9.9	63	12.8%	.62 [.21, 1.03]
Easton et al. (2008)	“”	16.5	8.5	9	9.33		3.1	7	2.6%	1.01 [−.06, 2.07]
Romero-Martínez et al. (2019b)	“”	27.14	17.67	89	14.03		13.73	39	13.7%	.79 [.40, 1.17]
Romero-Martínez et al. (2019c)	“”	27.87	15.1	63	13.7		13.46	39	12.3%	.97 [.55, 1.39]
Romero-Martínez et al. (2021a)	“”	25.28	18.35	47	13.7		13.46	41	11.9%	.71 [.27, 1.14]
Romero-Martínez et al. (2022a, b)	“”	25.4	16.78	120	14.5		13.33	82	19.1%	.70 [.41, .99]
Stanford et al. (2007)	“”	14.3	5.6	18	13.9		6.9	18	6.3%	.06 [−.59, .72]
Vitoria-Estruch et al. (2018)	“”	27.83	17.59	35	13.67		13.75	37	10.1%	.89 [.40, 1.38]
Westby and Ferraro (1999)	“”	16.57	11.16	38	14.63		10.48	38	11.2%	.18 [−.27, .63]
Total (95% CI)				458				364	100.0%	.66 [.48, .84]
Heterogeneity: Tau^2^ = .02; Chi^2^ = 11.50, df = 8 (*p* = .17); *I*^2^ = 30%; Test for overall effect: *Z* = 7.24 (*p* < .00001)										
(b)										
**Study**	**Test**		**IPV perpetrators + drugs**			**Controls**			**Weight**	**Std. mean difference**
			**Mean**	**SD**	**Sample size**	**Mean**	**SD**	**Sample size**		**IV, random, 95% CI**
Easton et al. (2008)	WCST perseverative errors		11.44	3.8	9	9.33	3.1	7	22.4%	.57 [−.45, 1.58]
Romero-Martínez et al. (2013a, b)	“”		13.78	4.37	17	1.95	0.36	19	21.0%	3.84 [2.70, 4.99]
Romero-Martínez et al. (2022a, b)	“”		24.4	16.78	104	14.5	13.33	82	29.1%	.64 [.35, .94]
Vitoria-Estruch et al. (2018)	“”		25.68	11.47	28	13.67	13.75	37	27.5%	.93 [.41, 1.44]
Total (95% CI)					158			145	100.0%	1.38 [.41, 2.34]
Heterogeneity: Tau^2^ = .81; Chi^2^ = 28.48, df = 3 (*p* < .00001); *I*^2^ = 89%; Test for overall effect:* Z* = 2.80 (*p* = .005)										

In addition, when we focused on IPV perpetrators with drug misuse, these men also committed more perseverative errors than non-violent men (4 studies: SMD = 1.38, 95% CI [0.41, 2.34]; test for overall effect: *Z* = 2.80 (*p* < 0.005), but an important variability among included studies was found (Heterogeneity: Tau^2^ = 0.81, Chi^2^ = 28.48, df = 3 (*p* = 0.00001); *I*^2^ = 89%) (Table 4b).

The difference in the comparison between IPV perpetrators without drug misuse and non-violent men groups was moderate, given that their values were between 0.40 and 0.80. This difference was larger for the second case; the obtained values were higher than 0.80, although this effect was conditioned by an important heterogeneity across studies.

##### Planning Abilities

The calculation of differences in terms of planning abilities of IPV perpetrators compared to non-violent men revealed a significant effect (3 studies: SMD = –0.63, 95% CI [−0.86, −0.41]; test for overall effect: *Z* = 5.51, *p* < 0.00001), with this conclusion being homogeneous across studies (Heterogeneity: Tau^2^ = 0.00, Chi^2^ = 1.24, *p* = 0.54); *I*^2^ = 0%) (Table 5a).

**Table 5:** Planning abilities in IPV perpetrators without (a) and with drug misuse (b) compared to non-violent men

**(a)**												
**Study**	**Test**		**IPV perpetrators**			**Controls**					**Weight**	**Std. mean difference**
			**Mean**	**SD**	**Sample size**	**Mean**	**SD**		**Sample size**			**IV, random, 95% CI**
Cohen et al. (2003)	Porteus Maze Test total quotient		104.5	14.0	41	109.2	10.1		20		15.7%	−.36 [−.90, .18]
Romero-Martínez et al. (2022a, b)	Key test (BADS) total score		8.44	3.84	120	11.16	3.81		82		54.5%	−.71 [−1.00, −.42]
Vitoria-Estruch et al. (2018)	Key test (BADS) total score		7.59	6.35	35	10.92	3.8		37		20.3%	−.63 [−1.11, −.16]
Total (95% CI)					196				139		100.0%	−.63 [−.86, −.41]
Heterogeneity: Tau^2^ = .00; Chi^2^ = 1.24, df = 2 (*p* = .54); *I*^2^ = 0%; Test for overall effect: *Z* = 5.51 (*p* < .00001)												
(b)												
**Study**		**Test**		**IPV perpetrators + drugs**			**Controls**				**Weight**	**Std. Mean difference**
				**Mean**	**SD**	**Sample size**	**Mean**	**SD**		**Sample size**		**IV, Random, 95% CI**
Romero-Martínez et al. (2019c)		Key test (BADS) total score		7.84	3.59	63	11.21	3.58		39	27.5%	−.93 [−1.35, −.51]
Romero-Martínez et al. (2022a, b)		“”		8.35	3.83	104	11.16	3.81		82	54.3%	−.73 [−1.03, −.43]
Vitoria-Estruch et al. (2018)		“”		6.63	5.47	28	10.92	3.8		37	18.1%	−.92 [−1.44, −.41]
Total (95% CI)						195				158	100.0%	−.82 [−1.04, −.60]
Heterogeneity: Tau^2^ = .00; Chi^2^ = .76, df = 2 (*p* = .68); *I*^2^ = 0%; Test for overall effect: *Z* = 7.31 (*p* < .00001)												

This difference between groups was also present after comparing IPV perpetrators with drug misuse and non-violent men (3 studies: SMD = −0.82, 95% CI [−1.04, −0.60]; test for overall effect: *Z* = 7.31, *p* < 0.00001). Furthermore, heterogeneity assessment did not reveal a significant effect (Heterogeneity: Tau^2^ = 0.00, Chi^2^ = 0.76, *p* = 0.68); *I*^2^ = 0%) (Table 5b).

In both cases, IPV perpetrators presented lower planning abilities than non-violent men. Differences between groups in the first case could be defined as moderate and large in the second case.

##### Decision-Making

The analysis of differences in decision-making processes between IPV perpetrators without drug misuse and non-violent men did not reveal a significant effect (4 studies: SMD = **−**0.17, 95% CI [−0.84, 0.51]; test for overall effect: *Z* = 0.48, *p* = 0.63). In addition, heterogeneity assessment revealed a significant variation across groups (Heterogeneity: Tau^2^ = 0.37, Chi^2^ = 16.88, *p* = 0.0007); *I*^2^ = 82%) (Table 6a).

**Table 6:** Decision-making in IPV perpetrators without (a) and with drug misuse (b) compared to non-violent men

**(a)**									
**Study**	**Test**	**IPV perpetrators**			**Controls**			**Weight**	**Std. mean difference**
		**Mean**	**SD**	**Sample size**	**Mean**	**SD**	**Sample size**		**IV, random, 95% CI**
Amaoui et al. (2022)	IGT total score	−1.31	17.49	26	8.62	22.79	29	26.9%	−.48 [−1.02, .06]
Easton et al. (2008)	IGT total score	−5.33	21.0	9	22.0	6.09	7	16.4%	−1.58 [−2.75, -.41]
Romero-Martínez et al. (2021b)	CGT risk taking	.65	.19	51	.55	.17	39	28.7%	.55 [.12, .97]
Vitoria-Estruch et al. (2018)	CGT risk taking	.58	.17	35	.54	.17	37	28.1%	.23 [−.23, .70]
Total (95% CI)				121			112	100.0%	−.17 [−.84, .51]
Heterogeneity: Tau^2^ = .37; Chi^2^ = 16.88, df = 3 (*p* = .0007); *I*^2^ = 82%; test for overall effect: *Z* = .48 (*p* = .63)									
(b)									
**Study**	**Test**	**IPV perpetrators + drugs**			**Controls**			**Weight**	**Std. mean difference**
		**Mean**	**SD**	**Sample size**	**Mean**	**SD**	**Sample size**		**IV, random, 95% CI**
Easton et al. (2008)	IGT total score	−7.33	9.07	9	22.0	6.09	7	32.9%	−3.50 [−5.21, −1.79]
Romero-Martínez et al. (2019c)	CGT risk taking	.62	.16	63	19.39	3.84	39	33.4%	−7.86 [−9.03, −6.69]
Vitoria-Estruch et al. (2018)	CGT risk taking	.66	.13	28	.54	.17	37	33.7%	.77 [.26, 1.28]
Total (95% CI)				100			83	100.0%	−3.51 [−9.42, 2.40]
Heterogeneity: Tau^2^ = 26.89; Chi^2^ = 185.78, df = 2 (*p* < .00001); *I*^2^ = 99%; test for overall effect: *Z* = 1.17 (*p* = .24)									

This was the same for the comparison between IPV perpetrators with drug misuse and non-violent men (3 studies: SMD = −3.51, 95% CI [−9.42, 2.40]; test for overall effect: *Z* = 1.17, *p* = 0.24). Heterogeneity measurements revealed a significant effect (Heterogeneity: Tau^2^ = 26.89, Chi^2^ = 185.78, *p* = 0.00001); *I*^2^ = 99%) (Table 6b).

##### Phonemic Fluency

For the phonemic fluency, there was a significant difference between IPV perpetrators without drug misuse and non-violent men (3 studies: SMD = −0.69, 95% CI [−1.27, −0.10]; test for overall effect: *Z* = 2.31 (*p* = 0.02). This effect was relatively moderate as it did not reach an average of 0.80. However, the results vary across studies (Heterogeneity: Tau^2^ = 0.22, Chi^2^ = 12.32, *p* = 0.002); *I*^2^ = 84%) (Table 7a).

**Table 7:** Phonemic fluency in IPV perpetrators without (a) and with drug misuse (b) compared to non-violent men

**(a)**									
**Study**	**Test**	**IPV perpetrators**			**Controls**			**Weight**	**Std. mean difference**
		**Mean**	**SD**	**Sample size**	**Mean**	**SD**	**Sample size**		**IV, random, 95% CI**
Cohen et al. (1999)	FAS phonemic	41.4	10.4	39	45.4	11.2	63	33.8%	−.36 [−.77, .04]
Cohen et al. (2003)	FAS phonemic	37.5	10.4	41	42.3	11.2	20	29.9%	−.44 [−.99, .10]
Romero-Martínez et al. (2022a, b)	FAS phonemic	29.41	12.04	120	44.84	14.22	82	36.3%	−1.19 [−1.49, −.88]
Total (95% CI)				200			165	100.0%	−.69 [−1.27, −.10]
Heterogeneity: Tau^2^ = .22; Chi^2^ = 12.32, df = 2 (*p* = .002); *I*^2^ = 84%; test for overall effect: *Z* = 2.31 (*p* = .02)									
(b)									
**Study**	**Test**	**IPV perpetrators + drugs**			**Controls**			**Weight**	**Std. mean difference**
		**Mean**	**SD**	**Sample size**	**Mean**	**SD**	**Sample size**		**IV, random, 95% CI**
Romero-Martínez et al. (2021a)	FAS phonemic	33.91	14.89	47	40.37	13.82	41	48.2%	−0.44 [−0.87, −0.02]
Romero-Martínez et al. (2022a, b)	FAS phonemic	29.17	11.94	104	44.84	14.22	82	51.8%	−1.20 [−1.52, −0.89]
Total (95% CI)				151			123	100.0%	−.84 [−1.58, −.10]
Heterogeneity: Tau^2^ = .25; Chi^2^ = 7.88, df = 1 (*p* = .005); *I*^2^ = 87%; test for overall effect: *Z* = 2.21 (*p* = .03)									

This conclusion was the same after comparing the performance of IPV perpetrators with drug misuse and non-violent men in terms of phonemic fluency (2 studies: SMD = −0.84, 95% CI [−1.58, −0.10]; test for overall effect: *Z* = 2.21 (*p* = 0.03). In fact, this effect was larger. However, these conclusions vary across studies (Heterogeneity: Tau^2^ = 0.25, Chi^2^ = 7.88, *p* = 0.005); *I*^2^ = 87%) (Table 7b).

##### IQ

The IQ assessment revealed that IPV perpetrators without drug misuse presented a lower IQ than non-violent men (3 studies: SMD = −0.42, 95% CI [−0.67, −0.18]; test for overall effect: *Z* = 3.39, *p* = 0.0007), with this effect being low. This conclusion was homogeneous across studies (Heterogeneity: Tau^2^ = 0.00, Chi^2^ = 1.00, *p* = 0.61; *I*^2^ = 0%) Table 8a).

**Table 8:** IQ in IPV perpetrators without (a) and with drug misuse (b) compared to non-violent men

**(a)**									
**Study**	**Test**	**IPV perpetrators**			**Controls**			**Weight**	**Std. mean difference**
		**Mean**	**SD**	**Sample size**	**Mean**	**SD**	**Sample size**		**IV, random, 95% CI**
Cohen et al. (2003)	WAIS−R	94.9	15.2	41	100.7	11.0	20	20.4%	−.41 [−.95, .13]
Easton et al. (2008)	Shipley IQ	101.7	12.8	9	101.0	8.1	7	6.1%	.06 [−.93, 1.05]
Romero-Martínez et al. (2022a, b)	K−BIT	96.03	12.79	120	101.78	11.63	82	73.5%	−.46 [−.75, −.18]
Total (95% CI)			170			109		100.0%	−.42 [−.67, −.18]
Heterogeneity: Tau^2^ = .00; Chi^2^ = 1.00, df = 2 (*p* = .61); *I*^2^ = 0%; test for overall effect: *Z* = 3.39 (*p* = .0007)									
(b)									
**Study**	**Test**	**IPV perpetrators + drugs**			**Controls**			**Weight**	**Std. mean difference**
		**Mean**	**SD**	**Sample size**	**Mean**	**SD**	**Sample size**		**IV, random, 95% CI**
Easton et al. (2008)	Shipley IQ	86.6	11.3	9	101.0	8.1	7	31.4%	−1.35 [−2.48, −.23]
Romero-Martínez et al. (2022a, b)	K-BIT	96.49	12.06	104	101.78	11.63	82	68.6%	−.44 [−.74, −.15]
Total (95% CI)				113			89	100.0%	−.73 [−1.56, .10]
Heterogeneity: Tau^2^ = .24; Chi^2^ = 2.35, df = 1 (*p* = .13); *I*^2^ = 57%; test for overall effect: *Z* = 1.73 (*p* = .08)									

However, the IQ of IPV perpetrators with drug misuse did not differ from the IQ of non-violent men (2 studies: SMD = −0.73, 95% CI [−1.56, 0.10]; test for overall effect: *Z* = 1.73, *p* = 0.08), and this conclusion was consistent across studies (Heterogeneity: Tau^2^ = 0.24, Chi^2^ = 2.35, *p* = 0.13; *I*^2^ = 0%) (Table 8b).

##### IPV Perpetrators vs Other Types of Criminal Convictions

The assessment of IQ group differences between IPV perpetrators and other criminal history did not reveal significant differences in terms of IQ (3 studies: SMD = 0.01, 95% CI [−0.43, 0.44]; test for overall effect: *Z* = 0.03, *p* = 0.97) and inhibitory control (2 studies: SMD = −0.09, 95% CI [−0.45, 0.27]; test for overall effect: *Z* = 0.50, *p* = 0.62). These conclusions were consistent in both cases across studies (Heterogeneity: Tau^2^ = 0.00, Chi^2^ = 0.33, *p* = 0.57; *I*^2^ = 0% and Heterogeneity: Tau^2^ = 0.00; Chi^2^ = 0.31, *p* = 0.58; *I*^2^ = 0%; respectively). However, a significant difference between these groups was found for switching attention (3 studies: SMD = 0.47, 95% CI [0.17, 0.78]; Test for overall effect: *Z* = 3.06, *p* = 0.002), with IPV perpetrators presenting a lower performance than other criminal history. The difference was low, and the conclusion was homogeneous (Heterogeneity: Tau^2^ = 0.00; Chi^2^ = 0.89, *p* = 0.64; *I*^2^ = 0%) (Table 9).

**Table 9:** Cognitive performance (IQ; inhibitory control and switch attention) in IPV perpetrators compared to other criminal convictions

*IQ*									
**Study**	**Test**	**IPV perpetrators**			**Other criminal convictions**			**Weight**	**Std. mean difference**
		**Mean**	**SD**	**Sample size**	**Mean**	**SD**	**Sample size**		**IV, random, 95% CI**
Britton et al. (2010)	TONI-2 (non-verbal)	73.3	12.8	27	74.4	14.0	28	21.4%	−.08 [−.61, .45]
Bueso-Izquierdo et al. (2016)	K-BIT (matrix)	32.0	.73	28	33.29	5.78	35	23.0%	−.29 [−.79, .21]
Verdejo-Román et al. (2019)	K-BIT (matrix)	32.44	6.26	21	29.25	6.41	20	28.9%	.49 [−.13, 1.12]
Total (95% CI)				76			83	100%	.01 [−.43, .44]
Heterogeneity: Tau^2^ = .07; Chi^2^ = 3.82, df = 2 (*p* = .15); *I*^2^ = 48%; test for overall effect: *Z* = .03 (*p* = .97)									
*Inhibitory control*									
**Study**	**Test**	**IPV perpetrators**			**Controls**			**Weight**	**Std. mean difference**
		**Mean**	**SD**	**Sample size**	**Mean**	**SD**	**Sample size**		**IV, random, 95% CI**
Britton et al. (2010)	Stroop (interf)	48.4	9.4	27	50.3	9.3	29	19.6%	−.20 [−.73, .33]
Bueso-Izquierdo et al. (2016)	TMT 4 (interf)	52.0	10.17	28	51.94	11.53	35	21.9%	.01 [−.49, .50]
Total (95% CI)				55			64	100%	−.09 [−.45, .27]
Heterogeneity: Tau^2^ = .00; Chi^2^ = .31, df = 1 (*p* = .58); *I*^2^ = 0%; test for overall effect: *Z* = .50 (*p* = .62)									
*Switch attention*									
**Study**	**Test**	**IPV perpetrators**			**Controls**			**Weight**	**Std. mean difference**
		**Mean**	**SD**	**Sample size**	**Mean**	**SD**	**Sample size**		**IV, random, 95% CI**
Amaoui et al. (2022)	Spatial Stroop task (switch)	−78.14	66.06	26	−93.89	51.17	29	32.6%	.26 [−.27, .80]
Britton et al. (2010)	TMT B	235.5	158.2	27	158.3	86.9	28	31.5%	.60 [.06, 1.14]
Bueso-Izquierdo et al. (2016)	TMT 4	99.04	24.93	28	82.94	31.45	35	35.9%	.55 [.05, 1.06]
Total (95% CI)				81			92	100%	.47 [.17, .78]
Heterogeneity: Tau^2^ = .00; Chi^2^ = .89, df = 2 (*p* = .64); *I*^2^ = 0%; Test for overall effect: *Z* = 3.06 (*p* = .002)									

##### IPV Perpetrators Without Drug Misuse vs IPV Perpetrators with Drug Misuse

The analysis of group differences across studies did not reveal significant differences between IPV perpetrators without drug misuse and those with drug misuse in terms of working memory (*Z* = 1.06; *p* = 0.29), cognitive flexibility (*Z* = 1.26; *p* = 0.21), or IQ (*Z* = 1.14; *p* = 0.26). Even though conclusions across studies were consistent for working memory (Heterogeneity: Tau^2^ = 0.03; Chi^2^ = 4.48, df = 3 (*p* = 0.21); *I*^2^ = 33%), there was a considerable variability for studies measuring cognitive flexibility and IQ (Heterogeneity: Tau^2^ = 2.05; Chi^2^ = 166.51, *p* < 0.00001); *I*^2^ = 98 and heterogeneity: Tau^2^ = 0.14; Chi^2^ = 6.12, *p* = 0.05); *I*^2^ = 67%, respectively). However, groups differed in terms of switching attention (4 studies: SMD = −0.51, 95% CI [−0.71, −0.30]; *Z* = 4.83, *p* < 0.00001). IPV perpetrators without drug misuse outperformed those with drug misuse in tasks measuring switching attention. Included studies presented a certain homogeneity given that the heterogeneity measurement was not significant (Heterogeneity: Tau^2^ = 0.00; Chi^2^ = 1.48, *p* = 0.69; *I*^2^ = 0%) (Table 10).

**Table 10:** Cognitive performance (working memory, switch attention, cognitive flexibility, and IQ) in IPV perpetrators without drug misuse compared to IPV perpetrators with drug misuse

Switch attention									
**Study**	**Test**	**IPV perpetrators**			**IPV perpetrators + drugs**			**Weight**	**Std. mean difference**
		**Mean**	**SD**	**Sample size**	**Mean**	**SD**	**Sample size**		**IV, random, 95% CI**
Bueso-Izquierdo et al. (2019)	TMT B	92.62	35.07	36	114.0	47.8	37	19.3%	−.50 [−.97, −.04]
Easton et al. (2008)	TMT B	69.6	32.1	9	94.9	25.8	9	4.4%	−.83 [−1.80, .15]
Romero-Martínez et al. (2022a, b)	CPT-III errors	28.84	22.2	120	38.71	24.17	104	59.4%	−.43 [−.69, −.16]
Vitoria-Estruch et al. (2018)	AST switch cost	-134.42	123.81	35	−55.9	80.57	28	16.0%	−.73 [−1.24, −.21]
Total (95% CI)				200			178	100.0%	−.51 [−.71, −.30]
Heterogeneity: Tau^2^ = .00; Chi^2^ = 1.48, df = 3 (*p* = .69); *I*^2^ = 0%; test for overall effect: *Z* = 4.83 (*p* < .00001)									
Working memory									
**Study**	**Test**	**IPV perpetrators**			**IPV perpetrators + drugs**			**Weight**	**Std. mean difference**
		**Mean**	**SD**	**Sample size**	**Mean**	**SD**	**Sample size**		**IV, random, 95% CI**
Bueso-Izquierdo et al. (2019)	WAIS-IV letter number	9.95	2.56	37	8.61	3.05	38	24.8%	.47 [.01, .93]
Easton et al. (2008)	TMT B	1.3	0.54	9	2.0	1.6	9	7.8%	−.56 [−1.51, .39]
Romero-Martínez et al. (2022a, b)	CPT-III errors	14.05	4.19	120	13.78	3.89	104	45.1%	.07 [−.20, .33]
Vitoria-Estruch et al. (2018)	AST switch cost	7.91	3.23	35	7.26	2.47	28	22.2%	.22 [−.28, .72]
Total (95% CI)				201			179	100.0%	.15 [−.13, .43]
Heterogeneity: Tau^2^ = .03; Chi^2^ = 4.48, df = 3 (*p* = .21); *I*^2^ = 33%; test for overall effect: *Z* = 1.06 (*p* = .29)									
Cognitive flexibility									
**Study**	**Test**	**IPV perpetrators**			**IPV perpetrators + drugs**			**Weight**	**Std. mean difference**
		**Mean**	**SD**	**Sample size**	**Mean**	**SD**	**Sample size**		**IV, random, 95% CI**
Easton et al. (2008)	WCST perseverative errors	16.5	8.5	9	11.44	3.8	9	6.0%	.73 [−.23, 1.69]
Romero-Martínez et al. (2013b)	“”	1.95	0.36	71	13.78	4.37	74	7.9%	−3.76 [−4.30, −3.21]
Romero-Martínez et al. (2016)	“”	22.09	12.24	61	29.21	19.79	55	8.5%	−.44 [−.80, −.07]
Romero-Martínez et al. (2019a)	“”	27.18	18.54	329	29.63	20.06	94	17.5%	−.13 [−0.36, 0.10]
Romero-Martínez et al. (2022a, b)	“”	25.4	16.78	120	24.4	16.78	104	8.8%	.06 [−.20, .32]
Vitoria-Estruch et al. (2018)	“”	27.83	17.59	35	25.68	11.47	28	20.2%	.14 [−.36, .64]
Total (95% CI)				296			270	100%	−.67 [−1.95, .61]
Heterogeneity: Tau^2^ = 1.20; Chi^2^ = 171.06, df = 5 (*p* < .00001); *I*^2^ = 97%; Test for overall effect: *Z* = 1.26 (*p* = .21)									
IQ									
**Study**	**Test**	**IPV perpetrators**			**IPV perpetrators + drugs**			**Weight**	**Std. mean difference**
		**Mean**	**SD**	**Sample size**	**Mean**	**SD**	**Sample size**		**IV, random, 95% CI**
Bueso-Izquierdo et al. (2019)	K-BIT	96.39	11.58	28	92.51	12.6	35	35.8%	.32 [−.19, .82]
Easton et al. (2008)	Shipley	101.7	12.8	9	86.6	11.3	9	17.7%	1.19 [0.17, 2.21]
Romero-Martínez et al. (2022a, b)	K-BIT	96.03	12.79	120	96.49	12.06	104	46.6%	−.04 [−.30, .23]
Total (95% CI)				296			270	100%	−.67 [−1.95, .61]
Heterogeneity: Tau^2^ = .14; Chi^2^ = 6.12, df = 2 (*p* = .05); *I*^2^ = 67%; Test for overall effect: *Z* = 1.14 (*p* = .26)									

#### Association Between Neuropsychological Performance and IPV Perpetration Measured with the Physical Assault and Psychological Aggression Subscales of the CTS or CTS2

Working memory performance, especially for visuospatial information, was negatively and significantly associated with physical IPV (*r* = −0.17, 95% CIs [−0.44, −12]; *Z* = −2.52, *p* = 0.012), with this conclusion being homogeneous (Tau^2^ = 0.00; *Q* = 2.72, *p* = 0.26; *I*^2^ = 26.58%). However, working memory was not related to psychological IPV (*r* = −0.09, 95% CIs [−0.37, 0.21]; *Z* = −1.24, *p* = 0.207). These results were consistent across studies (Tau^2^ = 0.00; *Q* = 2.86, *p* = 0.24; *I*^2^ = 30.19%) (Table 11).

**Table 11:** Relationships between working memory with physical and psychological IPV in IPV perpetrators

**Working memory**																		
**Study**	**Test**			**Physical IPV**									**Psychological IPV**					
				**Sample size**					***r***	**95% CI**		**Weight**	***r***	**95% CI**			**Weight**	
Chiu et al. (2022)	ANAM subs			217					−.18**	[−.31, −.05]		52.21%	−.12	[−.25, .01]			51.18%	
Godfrey et al. (2020)	CorsiBlock Tapping Task			49					−.34**	[−.57, −.06]		17.24%	−.22	[−.50, .06]			17.85%	
Schumacher et al. (2013)	Symbol Digit Modalities Test			97					−.06	[−.26, .14]		30.55%	.05	[−.15, .25]			30.97%	
				363					−.17	[−.44, 12]		100%	−.09	[−.37, .21]			100%	
Heterogeneity: Tau^2^ = .00; *Q* = 2.72, *p* = .26; *I*^2^ = 26.58%; test for overall effect: *Z* = −2.52, *p* = .012 Heterogeneity: Tau^2^ = .00; *Q* = 2.86, *p* = .24; *I*^2^ = 30.19%; test for overall effect: *Z* = −1.24, *p* = .207																		
**Switch attention**																		
**Study**	**Test**		**Physical IPV**										**Psychological IPV**					
			**Sample size**			***r***				**95% CI**		**Weight**	***r***	**95% CI**			**Weight**	
Chiu et al. (2022)	TMT B-A		217			.09				[−.04, .22]		69.48%	.19**	[−.16, .10]			56.62%	
Schumacher et al. (2013)	TMT B		97			.03				[−.17, .23]		30.52%	−.02	[−.17, .23]			43.38%	
			314			.07				[−.27, .40]		100%	.10	[−.84, 89]			100%	
Heterogeneity: Tau^2^ = .00; *Q* = .24, *p* = .63; *I*^2^ = 0%; test for overall effect: *Z* = 2.59, *p* = .010 Heterogeneity: Tau^2^ = .00; *Q* = 2.94, *p* = .09; *I*^2^ = 66.04%; test for overall effect: *Z* = .95, *p* = .341																		
**Continuous attention**																		
**Study**	**Test**		**Physical IPV**										**Psychological IPV**					
			**Sample size**			***r***				**95% CI**		**Weight**	***r***	**95% CI**			**Weight**	
Chiu et al. (2022)	CPT		217			−.07				[−.20, .06]		55.58%	−.03	[−.16, .10]			50.45%	
Persampiere et al. (2014)	Go No Go		80			.00				[−.22, .22]		20%	−.20	[−.41, .01]			22.69%	
Schumacher et al. (2013)	Go Stop		97			−.03				[−.23, .17]		24.42%	.03	[−.17, .23]			26.86%	
			394			−.05				[−.13, .04]		100%	−.05	[−.30, 20]			100%	
Heterogeneity: Tau^2^ = .00; *Q* = .31, *p* = .86; *I*^2^ = 0%; test for overall effect: *Z* = −2.30, *p* = .021 Heterogeneity: Tau^2^ = .00; *Q* = 2.48, *p* = .29; *I*^2^ = 19.47%; test for overall effect: *Z* = −.88, *p* = .377																		
**Cognitive flexibility**																		
**Study**		**Test**		**Physical IPV**									**Psychological IPV**					
				**Sample size**			***r***			**95% CI**		**Weight**	***r***	**95% CI**			**Weight**	
Persampiere et al. (2014)		WCST		80			−.02			[−.24, .20]		45.03%	.02	[−.20, .24]			45.03%	
Schumacher et al. (2013)		Category Test Computer Version total		97			−.05			[−.25, .15]		54.97%	−.13	[−.32, .07]			54.97%	
				177			−.04			[−.22, .15]		100%	−.06	[−.77, 71]			100%	
Heterogeneity: Tau^2^ = .00; *Q* = .04, *p* = .85; *I*^2^ = 0%; test for overall effect: *Z* = 2.44, *p* = .015 Heterogeneity: Tau^2^ = .00; *Q* = .96, *p* = .33; *I*^2^ = 0%; test for overall effect: *Z* = −.84, *p* = .402																		
**Planning abilities**																		
**Study**		**Test**			**Physical IPV**								**Psychological IPV**					
					**Sample size**			***r***			**95% CI**		**Weight**	***r***	**95% CI**			**Weight**
Persampiere et al. (2014)		Tower of London			80			−.09			[−.31, .14]		45.03%	−.03	[−.25, .19]			45.03%
Schumacher et al. (2013)		Tower of London			97			.05			[−.15, .25]		54.97%	−.04	[−.24, .16]			54.97%
					177			−.01			[−.72, .70]		100%	−.04	[−.10, 03]			100%
Heterogeneity: Tau^2^ = .00; *Q* = .83, *p* = .36; *I*^2^ = 0%; test for overall effect: *Z* = −.19, *p* = .851 Heterogeneity: Tau^2^ = .00; *Q* = .00, *p* = .94; *I*^2^ = 0%; test for overall effect: *Z* = −7.13, *p* < .001																		
**Inhibitory control**																		
**Study**	**Test**				**Physical IPV**								**Psychological IPV**					
					**Sample size**			***r***			**95% CI**		**Weight**	***r***		**95% CI**		**Weight**
Persampiere et al. (2014)	Stroop Interference				80			−.06			[−.28, .17]		45.03%	−.11		[−.33, .12]		45.96%
Schumacher et al. (2013)	Stroop Total Score				97			.05			[−.15, .25]		54.97%	.06		[−.14, .26]		54.04%
					177			.00			[−.60, .60]		100%	−.02		[−.80, 79]		100%
Heterogeneity: Tau^2^ = .00; *Q* = .51, *p* = .47; *I*^2^ = 0%; test for overall effect: *Z* = .01, *p* = .993 Heterogeneity: Tau^2^ = .00; *Q* = 1.23, *p* = .27; *I*^2^ = 28.75%; test for overall effect: *Z* = −.22, *p* = .829																		

The analysis of switching attention and IPV perpetration revealed that it was not related to physical IPV (*r* = 0.07, 95% CIs [−0.27, 0.40]; *Z* = −2.59, *p* = 0.010), with this conclusion being homogeneous (Tau^2^ = 0.00; *Q* = 0.24, *p* = 0.63; *I*^2^ = 0%). This was the same for the analysis of the association between switching attention and psychological IPV (*r* = 0.10, 95% CIs [−0.84, 0.89]; *Z* = 0.95, *p* = 0.341), with this result being explained by the heterogeneity across studies (Tau^2^ = 0.00; *Q* = 2.94, *p* = 0.09; *I*^2^ = 66.04%) (Table 11).

Continuous attention performance was weakly but significantly associated with physical IPV (*r* = −0.05, 95% CIs [−0.13, 0.04]; *Z* = −2.30, *p* = 0.021), with this conclusion being homogeneous (Tau^2^ = 0.00; *Q* = 0.31, *p* = 0.86; *I*^2^ = 0%). However, it was not related to psychological IPV (*r* = −0.05, 95% CIs [−0.30, 0.20]; *Z* = −0.88, *p* = 0.377), which was a consistent conclusion across studies (Tau^2^ = 0.00; *Q* = 2.48, *p* = 0.29; *I*^2^ = 19.47%) (Table 11).

The analysis of cognitive flexibility performance and physical IPV revealed a non-significant association (*r* = −0.04, 95% CIs [−0.22, 0.15]; *Z* = 2.44, *p* = 0.015), which was homogeneous across studies (Tau^2^ = 0.00; *Q* = 0.04, *p* = 0.85; *I*^2^ = 0%). This was similar for the association with psychological IPV (*r* = −0.06, 95% CIs [−0.77, 0.71]; *Z* = −0.84, *p* = 0.402), which was consistent (Tau^2^ = 0.00; *Q* = 0.96, *p* = 0.33; *I*^2^ = 0%) (Table 11).

Planning abilities were not related to physical and psychological IPV (*r* = −0.01, 95% CIs [−0.72, 0.70]; *Z* = −0.19, *p* = 0.851; and *r* = −0.04, 95% CIs [−0.10, 0.03]; *Z* = −7.13, *p* < 0.001, respectively). This conclusion was homogeneous across studies (Tau^2^ = 0.00; *Q* = 0.83, *p* = 0.36; *I*^2^ = 0% and Tau^2^ = 0.00; *Q* = 0.00, *p* = 0.94; *I*^2^ = 0%, respectively) (Table 11).

The analysis of inhibitory control was not related to physical and psychological IPV (*r* = 0.00, 95% CIs [−0.60, 0.60]; *Z* = 0.01, *p* = 0.993 and *r* = −0.02, 95% CIs [−0.80, 0.79]; *Z* = −0.22, *p* = 0.829). This conclusion was homogeneous across studies (Tau^2^ = 0.00; *Q* = 0.51, *p* = 0.47; *I*^2^ = 0% and Tau^2^ = 0.00; *Q* = 1.23, *p* = 0.27; *I*^2^ = 28.75%) (Table 11).

## Discussion

As far as we known, this is the first meta-analysis to calculate differences between IPV perpetrators and other samples of men (non-violent men, IPV perpetrators with drug misuse, and other types of convicted men) based on their neuropsychological performance. Considering studies that compare IPV perpetrators with non-violent men (well-matched for demographic characteristics), our findings indicate that IPV perpetrators, regardless of their drug misuse, exhibit poorer neuropsychological functioning compared to non-violent men. These differences ranged from moderate to large in working memory, switching attention, cognitive flexibility, planning abilities, and phonemic fluency. The decision-making process did not differ between groups. Furthermore, whereas low functioning IQ was only observed in IPV perpetrators without drug misuse, continuous attention performance only differed in IPV perpetrators with drug misuse and non-violent men. The majority of these conclusions were consistent across studies. The other comparisons between IPV perpetrators and other samples revealed that IPV perpetrators exhibited worse switching attention than the rest of the subsamples (IPV perpetrators without drug misuse and other types of convicted men). We found some support for an association between working memory, switch and continuous attention, and cognitive flexibility performance and IPV physical perpetration. Furthermore, planning abilities were inversely associated with IPV psychological perpetration. That is, our data partly supported our initial hypothesis regarding a positive association between neuropsychological deficits and IPV perpetration.

Most of the scientific research included in this meta-analysis was cross-sectional research, except for a longitudinal study (Chiu et al., 2022) with relatively small sample sizes (*n* ≤ 50 per group) but well-matched control groups for socio-demographic samples. Studies mostly assessed Caucasian and Hispanic convicted men. As can be seen in Supplementary Table 1, research was conducted with different samples in different countries from 1999 to 2022, but approximately 54% of these studies were conducted by a small number of Spanish research teams during the last years (Bueso-Izquierdo et al., 2016, 2019; Romero-Martínez et al., 2013a, b, 2016, 2019a, b, 2021a, b, 2022a; Verdejo-Román et al., 2019; Vitoria-Estruch et al., 2018). The risk of bias assessment revealed that some of this research exhibited a moderate to high risk of bias according to the criteria we established. Particularly, 48% of these studies were classified as moderate risk of bias, followed by a 40% of those studies presenting a high risk of bias, and, finally, 12% of them were classified as low risk. This should be considered when conducting future empirical research in this field.

Regarding the first aim of this study, our findings corroborate and complement the existing evidence of neuropsychological impairments in IPV perpetrators (working memory, switching attention, cognitive flexibility, planning abilities, and phonemic fluency). These impairments were consistently observed across the studies included in our analysis when comparing IPV perpetrators to non-violent men (Horne et al., 2020; Humenik et al., 2020; Romero-Martínez & Moya-Albiol, 2013). However, this impaired functioning was not generalized given that the groups did not differ in terms of decision-making processes. Furthermore, two exceptions were revealed in terms of IQ (only differing in IPV perpetrators without drug misuse) and continuous attention (only differing in IPV perpetrators with drug misuse), with IPV perpetrators presenting worse scores than non-violent men in both cases. We can speculate that these differences could be attributed to methodological considerations, such as the criteria used to distinguish groups of IPV perpetrators. In this regard, the typology “absence of alcohol use disorder” did not entail the complete absence of alcohol consumption or other drugs. Therefore, it is important to investigate whether this criterion for identifying subgroups of IPV perpetrators was derived from a single instrument or a combination of several instruments or criteria that indicate the presence of this disorder.

It should be noted that even though differences between groups were slightly larger (effect size larger than 0.80) and consistent in IPV perpetrators with drug misuse, these were also moderate in IPV perpetrators without drug misuse (effect sizes ranging from 0.04 to 0.08). Except for phonemic fluency, these effects were not affected by the heterogeneity across studies. Therefore, we cannot conclude that drugs are the main cause of neuropsychological functioning in IPV perpetrators, given that those differences appeared even in individuals without drug misuse. The conclusions of this meta-analysis have significant implications for current research. They highlight the importance of considering neuropsychological functioning as a potential risk factor for IPV perpetration, independent of drug misuse. This does not mean that they are completely independent factors, but drug misuse might accentuate differences between IPV perpetrators and controls.

Even though many of the studies included in this meta-analysis were cross-sectional and did not offer too much information regarding mediating factors that explain cognitive functioning, it would be interesting to explore whether cognitive remediation or training could impact the above-mentioned variables. In this regard, a recent randomized controlled trial was conducted (Romero-Martínez et al., 2022b). This trial administered a cognitive training to IPV perpetrators and analyzed the impact of worse cognitive functioning. The study concluded that the training had a positive impact on their performance, which in turn, also reduced future risk of recidivism. Therefore, a deeper understanding of IPV perpetrators’ cognitive needs would increase our ability to design adequate coadjutant cognitive treatments (i.e., cognitive training) to support standard batterer interventions. IPV perpetrators’ worse functioning in the above-mentioned cases does not necessarily indicate a functioning under the 50th percentile or normality. That is, based on raw data (or scores obtained directly from a neuropsychological test), we cannot state or qualify those scores as “dysfunction” or below the 50th percentile. All the employed neuropsychological tests allow transforming raw scores into standard scores, which guarantee knowing their place in a percentile rank (e.g., ≤ 50th percentile). However, none of the included studies in this meta-analysis provided the percentile of their participants. Maybe IPV perpetrators’ performance was slightly worse than control groups but was still located within the average performance of the population. Thus, this should be addressed in future empirical studies given that this knowledge would directly affect clinical practice (e.g., knowing the therapeutic needs of these men).

Unfortunately, this meta-analysis also indicated that there was insufficient information regarding other cognitive domains. For example, the majority of research measured working memory employing digits, letters, or visuospatial information (Amaoui et al., 2022; Easton et al., 2008; Romero-Martínez et al., 2021a, 2022a) but neglected other types of stimulus such as words. Furthermore, it would be important to incorporate tests assessing long-term memory after a long period of time (25–30 min approximately), recall, learning efficiency, inhibitory control, abstract reasoning, among others. Some of these processes were assessed in one study, which considered different modalities of long-term memory (Vitoria-Estruch et al., 2018), so it was not possible to calculate effect size. This should be considered when conducting empirical research including broader neuropsychological sets to assess IPV perpetrators’ cognitive functioning.

It seems that the differences between subgroups of IPV perpetrators (categorized based on their drug misuse) and other types of criminal history were overstated, given that this meta-analysis only reflected differences in terms of switching attention in both cases, with IPV perpetrators performing worse in this cognitive domain. Even though the impact of this cognitive ability in the processing of emotional stimulus, such as emotion decoding abilities, would be particularly interesting, we considered that a differentiation based on “type of conviction” or “drug presence” did not allow establishing differences between those men. Thus, it might be necessary to include additional characteristics (e.g., personality traits, psychopathology) to establish differentiated groups.

Regarding the second aim of this meta-analysis, we tried to answer whether neuropsychological performance directly impacts or explains IPV perpetration. We initially pointed out that broader cognitive deficits lead to higher IPV proneness, which is in line with previous research (Golden et al., 1996; Richman et al., 1999; Sedgwick et al., 2017). This meta-analysis sustained, at least in part, the positive association between physical and psychological IPV and working memory, switching and continuous attention, cognitive flexibility, and planning abilities. Although all confidence intervals in the above-mentioned associations included zero, this could be interpreted as a high likelihood of obtaining an absence of relationship between both variables. However, even though we cannot state the direct association between neuropsychological functioning and at least two types of IPV perpetration, this does not deny their involvement. In any case, we consider the research measuring the impact of neuropsychological functioning on IPV perpetration to be particularly limited. In fact, the absence of significant associations between the majority of the variables might be explained by methodological issues, such as the low number of studies assessing it (only two per cognitive domain), the reduced number of cognitive domains included, or the type of test, employing only a self-report for assessing IPV instead of other indicators or victim reports, among others. Maybe to fully understand the impact of neuropsychological functioning on IPV perpetration, we should consider including other variables that directly impact behavioral regulation, for example, anger trait, emotional regulation, coping with stress, and alcohol intoxication (Birkley & Eckhardt, 2015; Eckhardt et al., 2021; Richard et al., 2022). Future empirical studies should address the limitations of the current research in this field. This can be achieved by incorporating a comprehensive set of neuropsychological tests that cover a wide range of cognitive domains. Additionally, it is important to use different reports that measure IPV perpetration, instead of relying solely on self-measurement.

This meta-analysis has some limitations inherent to the review and to the research studies included. Compared to systematic or literature reviews, a meta-analysis has to stick to empirical research that includes studies with published data or that will offer this data after asking for it. In addition, the second objective of this study focused on measuring the association of neuropsychological performance and IPV perpetration but neglected other types of relevant variables such as anger expression-out or trait, recidivism, antisocial behaviors, among others. This meant losing interesting conclusions in this field, although it also indicated the direction for future empirical research. Other important limitations include the lack of quantitative analyses assessing publication bias (eggers regression test, funnel plots, etc.) and how the conclusions can only be generalized to heterosexual men. Furthermore, other potential confounding variables, such as education, developmental disorders, post-traumatic stress disorder, cohort effects, and traumatic brain injury, among others, have not been studied which makes it difficult to fully understand the differences between groups. Another limitation might be the consensus among researchers for defining specific cognitive domains based on concrete neuropsychological tests. In fact, to assess cognitive flexibility, we only considered a subscale of the WCST without paying attention to other subscales. Conclusions might differ from this subscale. In other words, it is important to consider different tests to measure the same cognitive domain. Other limitations affected the second aim of this study, given that this conclusion was based on a small number of studies. This reduces the impact of the second objective to calculating the association between neuropsychological performance and IPV. In addition, IPV perpetration was only measured with the CTS or CTS2 but did not include other types of violence strategies (e.g., sexual coercion, reactive and proactive aggression). Last, many of the conclusions are based on a limited number of research teams with specific characteristics (i.e., Caucasian, Hispanics, low educational level), except for some cases which included African American (Chiu et al., 2022; Easton et al., 2008; Godfrey et al., 2020; Persampiere et al., 2014; Schumacher et al., 2013; Westby & Ferraro, 1999). Thus, it would be important for different researchers from other countries to conduct additional research with different samples, to attend to diversity. This would reinforce and strengthen the external validity of the conclusions derived from the studies. Finally, given that there are only a small number of studies available for meta-analysis (e.g., association between neuropsychological functioning and IPV) and the confidence interval around tau-square is large, there may be heterogeneity despite the results suggesting otherwise. However, it is difficult to accurately estimate this, so it would be important to increase the number of studies assessing this association to correct it.

In summary, this meta-analysis pointed out that IPV perpetrators tend to present a broad neuropsychological dysfunction or, at least, a slightly lower cognitive functioning when compared to controls, which affects attention, memory, and executive functioning, even without considering drug misuse. However, the differences between IPV perpetrator subtypes compared to other criminal history were overstated, given that they only differed significantly in switching attention. Moreover, these differences were consistent across studies, except for a few cognitive domains (e.g., decision making and phonemic fluency). These facts reinforce the need to establish forensic assessments including a set of neuropsychological tests to properly characterize the cognitive needs of these men before starting standard batterer interventions. Furthermore, we need to understand the impact of cognitive functioning on IPV perpetration. Unfortunately, the second aim of this study contradicted our initial hypothesis regarding the relevant impact of neuropsychological dysfunctions on IPV perpetration. One could argue that the cognitive domains that were identified do not directly relate to this type of violence. Therefore, it may be important to explore other factors that are more closely linked to behavioral regulation. In any case, we would like to reinforce the need for additional studies that measure the role of neuropsychological functioning in IPV perpetration by employing different instruments and measurements of this type of violence.

## Supplementary Information

Below is the link to the electronic supplementary material.

Supplementary file1 (DOCX 80 KB)

## Data Availability

All data and material used in this systematic review are available upon request.
