# Supplementary material for: A Meta-analysis of Cognitive Functioning in Intimate Partner Violence Perpetrators
Source: Neuropsychol Rev. 2023 Dec 12;34(4):1191–212. doi: 10.1007/s11065-023-09628-w (PMC11607039; doi:10.1007/s11065-023-09628-w)
Supplement: Supplementary file 1 — Supplementary file1 (DOCX 80 KB) [file 11065_2023_9628_MOESM1_ESM.docx]

| **Supplementary table 1**  *Summary of risk of bias for included studies in meta-analysis, alphabetically ordered by first author’s surname* | | | | | | | | | | | | | |  |
| --- | --- | --- | --- | --- | --- | --- | --- | --- | --- | --- | --- | --- | --- | --- |
| **Authors** | | **Criterion** | | | | | | | | | | | | **Risk of bias** |
|  |  | **Objectives** | | **Sample** | | | | **Procedure** | | | **Results** | | |  |
|  |  | 1a | 1b | 2a | 2b | 2c | 2d | 2e | 3a | 3b | 4a | 4b | 4c |  |
| Amaoui et al., 2022 | | Yes | Yes | No | No | Yes | No | No | Yes | Yes | Yes | No | Yes | Moderate |
| Britton et al., 2010 | | No | No | Yes | No | Yes | No | No | Yes | Yes | No | Yes | Yes | High |
| Bueso-Izquierdo et al., 2016 | | Yes | No | Yes | Yes | No | No | Yes | Yes | No | Yes | No | Yes | Moderate |
| Buesto-Izquierdo et al., 2019 | | Yes | No | Yes | Yes | No | No | No | Yes | No | Yes | Yes | No | High |
| Chiu et al., 2022 | | Yes | Yes | Yes | No | Yes | No | Yes | Yes | Yes | No | No | Yes | Moderate |
| Cohen et al., 1999 | | Yes | No | No | No | No | No | No | Yes | Yes | No | Yes | Yes | High |
| Cohen et al., 2003 | | No | No | No | Yes | No | No | No | Yes | No | No | Yes | Yes | High |
| Easton et al., 2008 | | No | No | Yes | Yes | No | No | No | Yes | No | Yes | Yes | No | High |
| Godfrey et al., 2020 | | Yes | Yes | Yes | No | No | No | No | Yes | Yes | No | No | Yes | High |
| Persampiere et al., 2014 | | Yes | No | No | No | No | No | Yes | Yes | Yes | No | Yes | Yes | High |
| Romero-Martínez et al., 2013a | | Yes | Yes | Yes | Yes | No | No | No | Yes | Yes | No | No | Yes | High |
| Romero-Martínez et al., 2013b | | Yes | Yes | Yes | Yes | No | No | Yes | Yes | Yes | Yes | No | Yes | Moderate |
| Romero-Martínez et al., 2016 | | Yes | Yes | No | Yes | Yes | No | Yes | Yes | Yes | Yes | Yes | Yes | Low |
| Romero-Martínez et al., 2019a | | Yes | Yes | Yes | Yes | No | No | No | Yes | Yes | No | Yes | Yes | Moderate |
| Romero-Martínez et al., 2019b | | Yes | Yes | Yes | Yes | No | No | No | Yes | Yes | Yes | No | Yes | Moderate |
| Romero-Martínez et al., 2019c | | Yes | Yes | Yes | Yes | No | No | Yes | Yes | Yes | No | No | Yes | Moderate |
| Romero-Martínez et al., 2021a | | Yes | Yes | Yes | Yes | No | No | No | Yes | Yes | Yes | Yes | Yes | Moderate |
| Romero-Martínez et al., 2021b | | Yes | Yes | Yes | Yes | No | No | No | Yes | Yes | Yes | Yes | Yes | Moderate |
| Romero-Martínez et al., 2022 | | Yes | Yes | Yes | Yes | Yes | Yes | Yes | Yes | Yes | Yes | Yes | Yes | Low |
| Schumacher et al., 2013 | | Yes | No | Yes | - | Yes | No | No | Yes | Yes | No | Yes | Yes | Moderate |
| Stanford et al., 2007 | | No | Yes | Yes | No | Yes | No | No | Yes | No | No | No | No | High |
| Verdejo-Román et al., 2019 | | Yes | Yes | Yes | Yes | No | No | No | Yes | No | Yes | Yes | No | Moderate |
| Vitoria-Estruch et al., 2018 | | Yes | Yes | Yes | Yes | Yes | No | No | Yes | Yes | Yes | Yes | Yes | Low |
| Westby & Ferraro, 1999 | | Yes | Yes | No | Yes | No | No | No | Yes | No | Yes | Yes | Yes | Moderate |
| Wilson et al., 2017 | | No | No | Yes | - | No | No | No | Yes | No | - | No | No | High |

| **Supplementary table 2**  *Information about demographic characteristics, alcohol misuse and traumatic brain injuries of the samples included in this meta-analysis, alphabetically ordered by first author’s surname* | | | | | | | | | | | |  |
| --- | --- | --- | --- | --- | --- | --- | --- | --- | --- | --- | --- | --- |
| **Authors** | |  | **Clinical and demographic characteristics** | | | | | | | | | |
|  |  | **Age** | | **Years of education or educational level** | | **Civil status** | | **Alcohol misuse (percentage of consumers or average score)** | | | **Traumatic brain injuries (percentage of participants or number of head injuries)** | |
|  |  | **IPV perpetrators** | **Controls** | **IPV perpetrators** | **Controls** | **IPV perpetrators** | **Controls** | | **IPV perpetrators** | **Controls** | **IPV perpetrators** | **Controls** |
| Amaoui et al., 2022 | | 41.19 (9.71) | 38.28 (8.54) | 9.19 (4.30) | 9.55 (3.58) | - | - | | - | - | - | - |
| Britton et al., 2010 | | 41.4 (12.1) | 34.3 (8.1) | 8.7 (3.2) | 9.3 (3.4) | - | - | | - | - | - | - |
| Bueso-Izquierdo et al., 2016 | | 36.29 (9.73) | 36.69 (8.36) | 2.39 (1.85) | 3.14 (1.85) | - | - | | 0% | 0% | 0% | 0% |
| Buesto-Izquierdo et al., 2019 | | 38.79 (6.74) | 35.59 (7.95) | 69.2% basic | 48.7% basic | 51.3% divorced | 59.5% divorced | | 100% | 0% | 0% | 0% |
| Chiu et al., 2022 | | 35.92 (5.94) | - | 48.8% secondary | - | - | - | | 14.7% | - | 63.6% | - |
| Cohen et al., 1999 | | 33.00 (9.4) | 34.9 (9.8) | 12.9 (2.4) | 14.8 (2.2) | - | - | | 2.2 (1.9) drinks/week | 1.8 (1.3) drinks/week | 46.2% | 20.6% |
| Cohen et al., 2003 | | 32.9 (9.6) | 30.5 (10.7) | 12.2 (2.5) | 11.8 (3.3) | 58.5% married | 70% married | | 40% | 9.3% | 45.5% | 15.2% |
| Easton et al., 2008 | | From 18 to 55 | | 13.1 (2.6) | | - | | | 50% | 0% | - | - |
| Godfrey et al., 2020 | | 32.33 (9.56) | - | - | - | - | - | | - | - | - | - |
| Persampiere et al., 2014 | | 34.20 (10.48) | - | 11.48 (1.86) | - | - | - | | - | - | - | - |
| Romero-Martínez et al., 2013a | | 38.00 (3.13) | 35.80 (1.47) | - | - | - | - | | 4% | 0% alcohol | 0 | 0 |
| Romero-Martínez et al., 2013b | | 38.34 (10.47) | 41.67 (11.21) | 58% basic | 58% basic | 47% divorced | 48% married | | 100% | 0% alcohol | 0 | 0 |
| Romero-Martínez et al., 2016 | | 39.59 (9.70) | 42.21 (11.22) | 57% secondary | 50% secondary | 48% single/divorced | 47% single/divorced | | 100% | 0% alcohol | 0 | 0 |
| Romero-Martínez et al., 2019a | | 40.43 (11.92) | 38.89 (9.97) | 55% basic | 62% basic | 77% divorced | 76% divorced | | 100% | 0% alcohol | 0 | 0 |
| Romero-Martínez et al., 2019b | | 40.10 (11.05) | 41.89 (11.10) | 54% basic | 42% basic | 78% divorced | 63% divorced | | 4.13 (5.15) | 3.28 (2.61) | 0 | 0 |
| Romero-Martínez et al., 2019c | | 39.73 (10.72) | 41.72 (11.01) | 57% basic | 56% secondary | 64 divorced | 78 divorced | | 80% | 85% | 0 | 0 |
| Romero-Martínez et al., 2021a | | 38.61 (11.40) | 41.72 (11.01) | 72% basic | 87% basic | 51% single | 49% single | | 8.05 (7.40) | 3.24 (2.61) | 0 | 0 |
| Romero-Martínez et al., 2021b | | 41.72 (11.01) | 40.10 (10.90) | 59% basic | 50% basic | - | - | | 3.04 (3.45) | 3.24 (2.61) | 51% | 62% |
| Romero-Martínez et al., 2022 | | 40.08 (9.49) | 40.07 (9.17)  40.30 (10.59) | 52% basic | 45% secondary  46% secondary | - | - | | 8.39 (7.77) | 2.42 (2.23)  2.98 (2.54) | 0 | 0  0 |
| Schumacher et al., 2013 | | 33.48 (9.70) | - | 53.70% secondary | - | 52.60% married | - | | 100% | - | 1.55 (1.47) | - |
| Stanford et al., 2007 | | 35.6 (5.7) | 30.5 (8.9) | 13.4 (2.4) | 14.2 (1.6) | - | - | | 33% | 0 | 16% | 0 |
| Verdejo-Román et al., 2019 | | 38.38 (8.70) | 34.74 (8.76) | 9.62 (3.90) | 9.53 (2.46) | - | - | | - | - | - | - |
| Vitoria-Estruch et al., 2018 | | 40.21 (11.90) | 39.34 (9.83) IPV  41.75 (11.00)  Controls | 71.43% basic | 48.57% secondary  45.95% secondary | 46% separated | 42.86% separated  51.35% single | | 100% | 0  0 | 48.14% | 40%  32.43% |
| Westby & Ferraro, 1999 | | 33.34 (6.61) | 32.37 (6.98) | 45% college | 45% college | 37% divorced | 58% married | - | | - | - | - |
| Wilson et al., 2017 | | 40.21 (9.91) | | 52.9% basic | | - | - | - | | - | - | - |
